# Spotlight on the invasion of a carabid beetle on an oceanic island over a 105-year period

**DOI:** 10.1038/s41598-020-72754-5

**Published:** 2020-10-13

**Authors:** Marc Lebouvier, Philippe Lambret, Alexia Garnier, Peter Convey, Yves Frenot, Philippe Vernon, David Renault

**Affiliations:** 1 CNRS, EcoBio (Ecosystèmes, biodiversité, évolution) - UMR 6553, University of Rennes 1, Bâtiment 14A, 263 Avenue du Gal Leclerc, 35042 Rennes cedex, France; 2 Réserve Naturelle Nationale des Terres Australes Françaises, Rue Gabriel Dejean, 97410 Saint Pierre, Ile de la Réunion, France; 3 British Antarctic Survey, Natural Environment Research Council, High Cross, Madingley Road, Cambridge, CB3 0ET UK; 4 Institut Universitaire de France (IUF), 1 Rue Descartes, 75231 Paris Cedex 05, France

**Keywords:** Biodiversity, Invasive species, Population dynamics

## Abstract

The flightless beetle *Merizodus soledadinus,* native to the Falkland Islands and southern South America, was introduced to the sub-Antarctic Kerguelen Islands in the early Twentieth Century. Using available literature data, in addition to collecting more than 2000 new survey (presence/absence) records of *M. soledadinus* over the 1991–2018 period, we confirmed the best estimate of the introduction date of *M. soledadinus* to the archipelago, and tracked subsequent changes in its abundance and geographical distribution. The range expansion of this flightless insect was initially slow, but has accelerated over the past 2 decades, in parallel with increased local abundance. Human activities may have facilitated further local colonization by *M. soledadinus*, which is now widespread in the eastern part of the archipelago. This predatory insect is a major threat to the native invertebrate fauna, in particular to the endemic wingless flies *Anatalanta aptera* and *Calycopteryx moseleyi* which can be locally eliminated by the beetle. Our distribution data also suggest an accelerating role of climate change in the range expansion of *M. soledadinus*, with populations now thriving in low altitude habitats. Considering that no control measures, let alone eradication, are practicable, it is essential to limit any further local range expansion of this aggressively invasive insect through human assistance. This study confirms the crucial importance of long term biosurveillance for the detection and monitoring of non-native species and the timely implementation of control measures.

## Introduction

The contribution of anthropogenic activities to biological invasions is escalating rapidly and unrelentingly^1^, placing the introduction and spread of non-native organisms amongst the most important contemporary ecological and conservation themes^2,3^. Human-assisted biological invasions^4^ can be considered as a six-step continuum: (1) entrainment of living/viable specimens or propagules in their native range, (2) transport, (3) introduction (release) in a new area, (4) establishment, i.e. successful completion of the full life cycle in the new area, (5) sustained population increase at the introduction site(s) and (6) further geographic expansion from the introduction site^5–7^. In a wide range of taxa, studies have examined how and why non-native species have breached natural environmental barriers to spread (^8,9^; reviewed by^10^ for insects and arachnids). Studies assessing the level of invasiveness of non-native organisms and the invasibility of (micro)habitats^11–13^ have also been undertaken. However, empirical studies that document the early stages of biological invasions, i.e. the establishment and proliferation of non-native populations, and the early stages of subsequent local range expansion, are rare in cases of unintentional introductions^14,15^.

For many if not most unintentional insect introductions, it is challenging to properly document the (1) geographical origin, (2) initial site of introduction, (3) means and date of introduction, and (4) subsequent natural (i.e. not further human assisted) spread of the non-native species within the colonized area (but see the example of the gypsy moth *Lymantria dispar* (Linnaeus, 1758) (Lepidoptera: Erebidae) whose geographic spread is continuously monitored in the USA^16,17^). Often, the non-native organisms are only observed for the first time after their population densities have increased markedly^18,19^, and/or when they start to have economic impacts, limiting our capacities to better understand lag effects during invasions^19–22^. In the cases of the introduction of the ladybird *Harmonia axyridis* (Pallas, 1773) (Coleoptera: Coccinellidae) in Europe in 1964 and the wasp *Vespa velutina* Lepeletier, 1836 (Hymenoptera: Vespidae) in France in 2005, both their arrival and subsequent geographic expansion were detected and monitored (e.g.^23,24^). Yet, the confounding influences of multiple introduction sites, multiple introduction events at a single site^25^, and the invasive bridgehead effect^26^ can prohibit examining the biological and environmental drivers of each step of the invasion process.

Even if the number of databases reporting species occurrence is growing, biodiversity databases, and in particular those constructed from long-term observations reporting the fine-scale spatial distribution of non-native species are, to date, rare^27^. This is particularly true in continental areas, where monitoring the distribution of organisms across large geographical areas requires considerable and time-consuming effort. Instead, information on geographical distributions and sources of invasion (geographic profiling) are frequently obtained using an array of mathematical models^28–31^. In comparison with continental areas, oceanic islands provide tractable opportunities for conducting invasion ecology studies offering, *inter alia*, geographical isolation that limits invasion events from other landmasses, and limited overall terrestrial area that can aid the practicality of surveys. Amongst oceanic islands, the sub-Antarctic islands located remotely in the Southern Ocean are of particular interest. On several of these islands, good documentation of the presence and distribution of non-native flora and fauna is available, although detailed knowledge of introduction events remains very uneven^32–36^. The absence of extensive anthropogenic activities or impacts (e.g. urbanization, industrial development, agriculture, environmental pollution) makes sub-Antarctic islands fruitful model systems for studying biological invasions.

**Figure 1:**
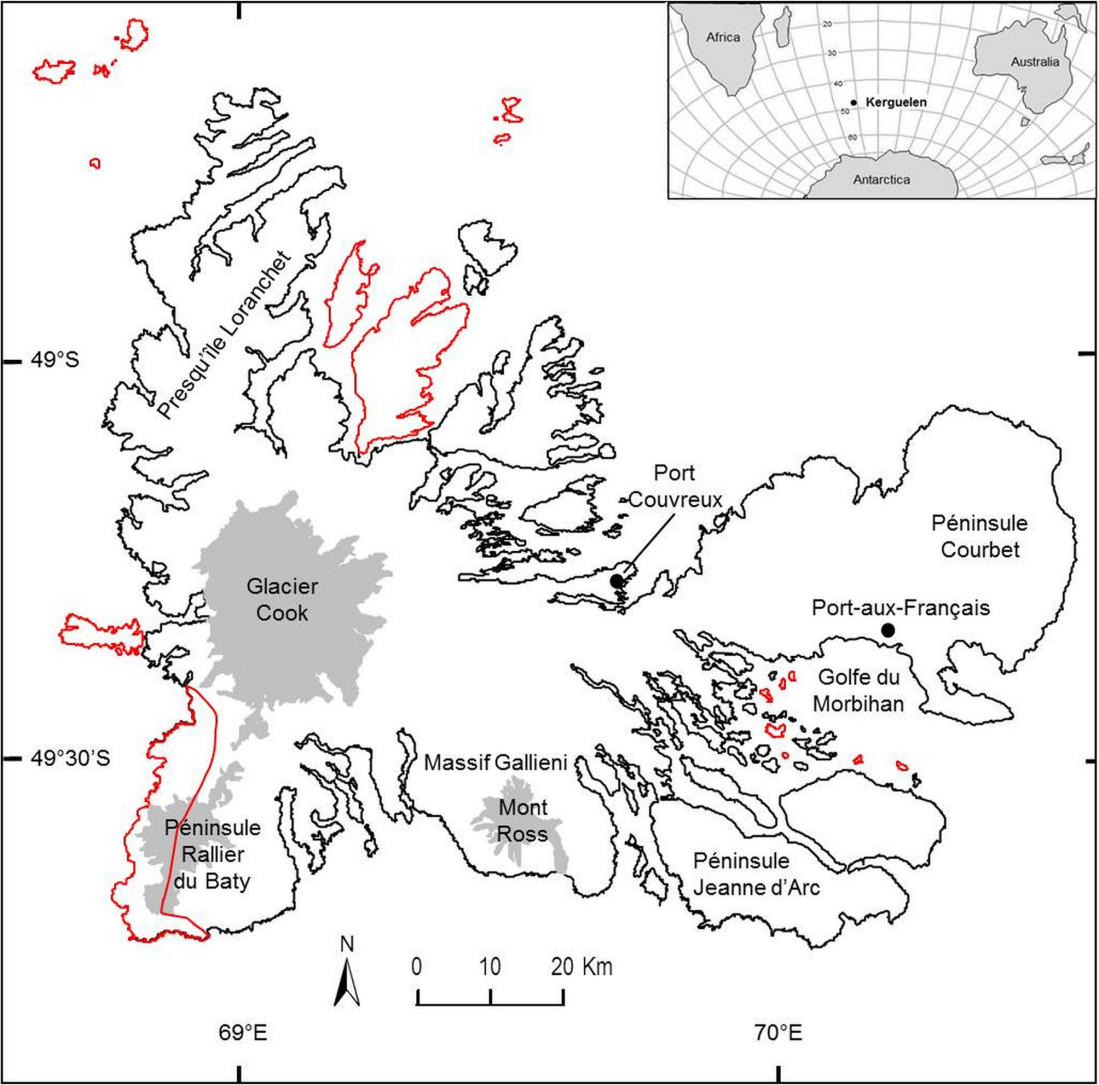
Location of the Kerguelen Islands in the Southern Hemisphere and map of the archipelago. All islands form a National Nature Reserve. Wilderness areas classified as “strict nature reserve” are indicated in red. Port-aux-Français is the research station and Port-Couvreux is an abandoned farm. The map of the Kerguelen Islands was created by the authors of the present study, and thus, the background map we used for the figure is not a copyrightable subject matter. Geospatial data were incorporated into the map with ArcMap in ArcGis 10.4 (https://www.esri.com), and this software was used to generate the map.

At the French sub-Antarctic Kerguelen Islands, the carabid beetle *Merizodus soledadinus* (Guérin-Méneville, 1830) (Coleoptera: Carabidae) was first observed in 1939^37^ (Fig. 1), and has subsequently spread to various parts of the archipelago^33,38,39^. As a predator of invertebrates, its geographic spread may have extreme impacts on available prey species. Higher trophic level invaders often have considerable ecological impacts, including driving biodiversity decline and the alteration of trophic webs. As the Kerguelen Islands are a recently declared UNESCO World Heritage Area and host many endemic insect species, the archipelago is of high biodiversity and conservation value. It is therefore vital to make first assessments of the possibly extreme impacts that *M. soledadinus* may have on available prey in the archipelago, where no equivalent native predators are present. Preliminary observations have revealed that this insect may threaten much of the native invertebrate fauna, in particular flightless native species such as the endemic flies *Anatalanta aptera* Eaton, 1875 (Diptera: Sphaeroceridae) and *Calycopteryx moseleyi* Eaton, 1875 (Diptera: Micropezidae). Such native invertebrates have very few natural predators or competitors and there is already evidence available that they have disappeared from some sites colonized by *M. soledadinus*^33,39^. Furthermore, as warming may accelerate the invasion front of *M. soledadinus*, it is crucial to evaluate the impact this predator can have on native species when establishing in new habitats.

In the present study, we provide a unique combination of (1) long-term monitoring retracing the invasion history and geographic expansion of the alien predator *M. soledadinus*, (2) field trapping to assess population dynamics and seasonal fluctuations from establishment onwards, and (3) targeted censuses of the density of the beetle and of two native flies known as form a significant part of its diet, to assess invasion dynamics and the resulting ecological effects on the native fauna of the Kerguelen Islands. To that end, we compiled evidence from the available literature on the history of ship visits and landings at the archipelago in order to provide a best estimate of the species’ introduction date. Then, by combining published information and long-term survey data from the archipelago, we report a 105-year time series documenting the range expansion of *M. soledadinus* since its introduction. The relative abundance, seasonal phenology, and ecological impacts of the beetle on *A. aptera* and *C. moseleyi* have been documented by trapping at two localities over 2 and 11 years. By taking advantage of this accidental and irreversible anthropogenic introduction, we present a unique study of the geographical spread and ecological impacts of a non-native predatory insect representing a new ecological guild in the ecosystems of the Kerguelen archipelago.

## Results

### Historical documentation of the invasion of the Kerguelen Islands by *Merizodus soledadinus*

*Merizodus soledadinus* was first observed on the Kerguelen Islands in February 1939 by Jeannel^37^ in the surroundings of the abandoned farm buildings of Port-Couvreux (Fig. 1), where he reported the presence of more than one thousand individuals. Our literature search identified 32 published documents^37,39–68^, including books, book chapters, dissertations, and primary literature, mentioning either the origin of the species, or its introduction and occurrence at the sub-Antarctic Kerguelen Islands and South Georgia.

### Temporal and spatial spread of *Merizodus soledadinus* in the Kerguelen Islands

**Figure 2:**
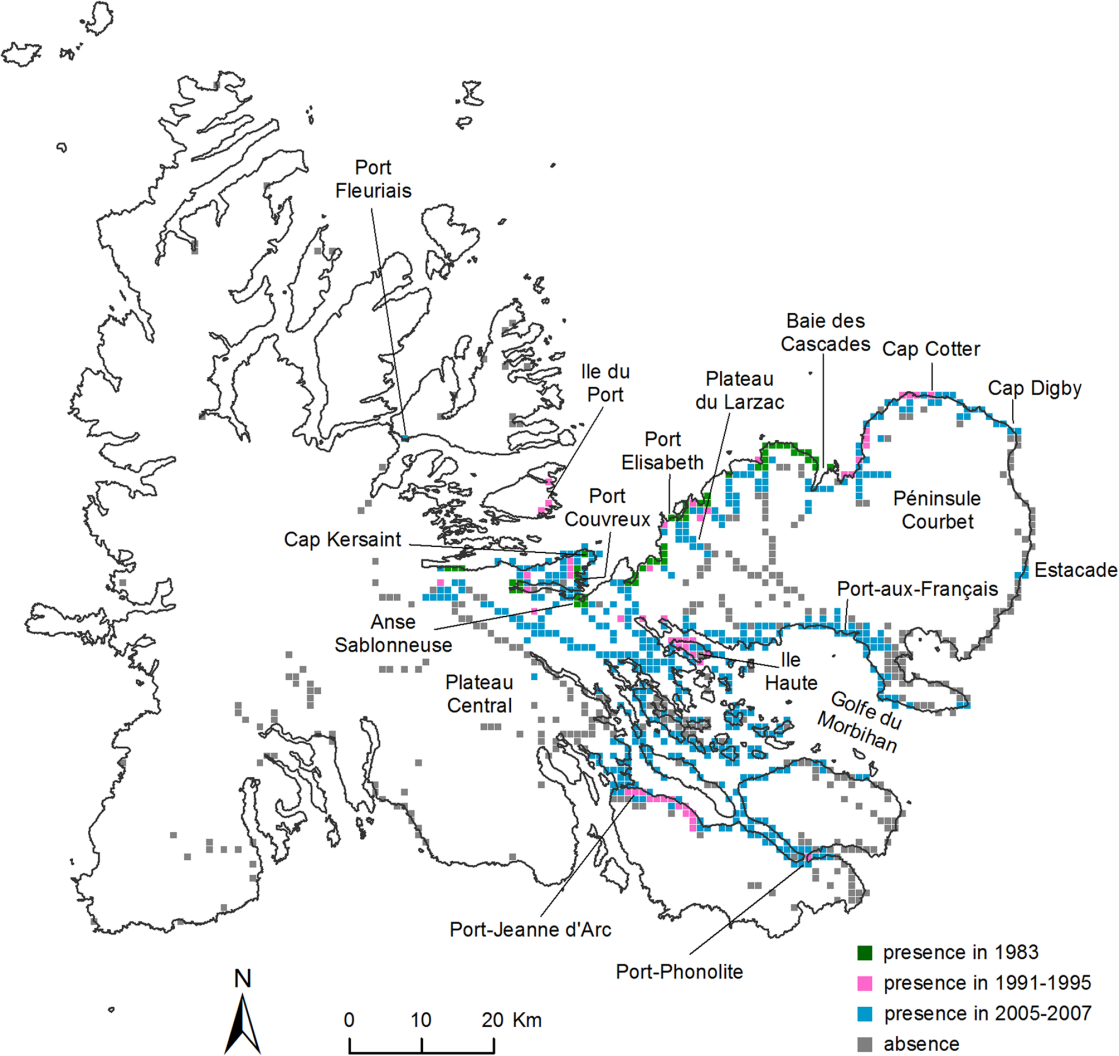
Distribution of *Merizodus soledadinus* on the Kerguelen Islands in 1982–1983 (after Dreux et al. 1992), between 1991 and 1995 (after Chevrier 1996), and between 2005 and 2007 (this study). The geographic occurrence squares report observations of the presence/absence of the insect; when present, the time ranges of these observations do not necessarily correspond to the establishment date of *M. soledadinus* at each of the surveyed localities. Observations are plotted on a one kilometer grid; coloured square = presence, grey square = absence. The map of the Kerguelen Islands was generated by the authors in ESRI ArcGis version 10.4 (www.esri.com). The background map we used for the figure is not a copyrightable subject matter.

After its initial introduction, the beetle remained restricted to the vicinity of the introduction site at Port-Couvreux for several decades^37^. In 1977, localities to the north of Port-Couvreux were colonized (Cap Kersaint, Presqu'Île Bouquet de la Grye)^54^. In 1982, Tréhen and Voisin^50^ reported the presence of *M. soledadinus* at Port Elisabeth (with a most probable establishment date around 1970), and it then colonized coastal habitats along the north-east coast of the Péninsule Courbet (Fig. 2) as far as the Baie des Cascades by 1983^54^; these localities were most probably colonised in the 1970s.

In the early 1990s, *M. soledadinus* expanded further along the Péninsule Courbet and reached Cap Cotter (Fig. 2)^42^. It also colonized an island close to Port-Couvreux (Île du Port), and further locations remote from its original point of introduction, Port-Phonolite and Port-Jeanne d’Arc, the latter the location of a historical whaling station that is regularly visited by both scientists and tourists, and Île Haute in the Golfe du Morbihan (Fig. 2). The species was first observed at the research station Port-aux-Français in 1999. At this site a monitoring programme using pitfall traps twice a month has run since 1996 for the documentation of terrestrial invertebrate communities. The beetle was first recorded in trap samples in June 2000, subsequently becoming common in the collections.

A large-scale survey conducted between 2005 and 2007 confirmed a considerable acceleration in the expansion of *M. soledadinus* in the archipelago (Fig. 2). By 2007, the insect was present along almost the entire coastline around the Golfe du Morbihan as well as on many islands in this bay, including islets that are rarely visited. Specimens were also reported from several additional sites on the main island of the archipelago including Port-Fleuriais (north of Port-Couvreux) and, on the east coast, in the vicinity of an isolated field hut (Estacade). Of particular note, the beetle was recorded for the first time inland, in vegetation along rivers (e.g. Gave de l’Azorella) and from low altitude fell-fields, a widespread sub-Antarctic habitat populated by cushion plants (Plateau du Larzac, in 2005, at 290 m above sea level (asl); Plateau Central, in 2006, five records between 278 and 358 m asl).

**Figure 3:**
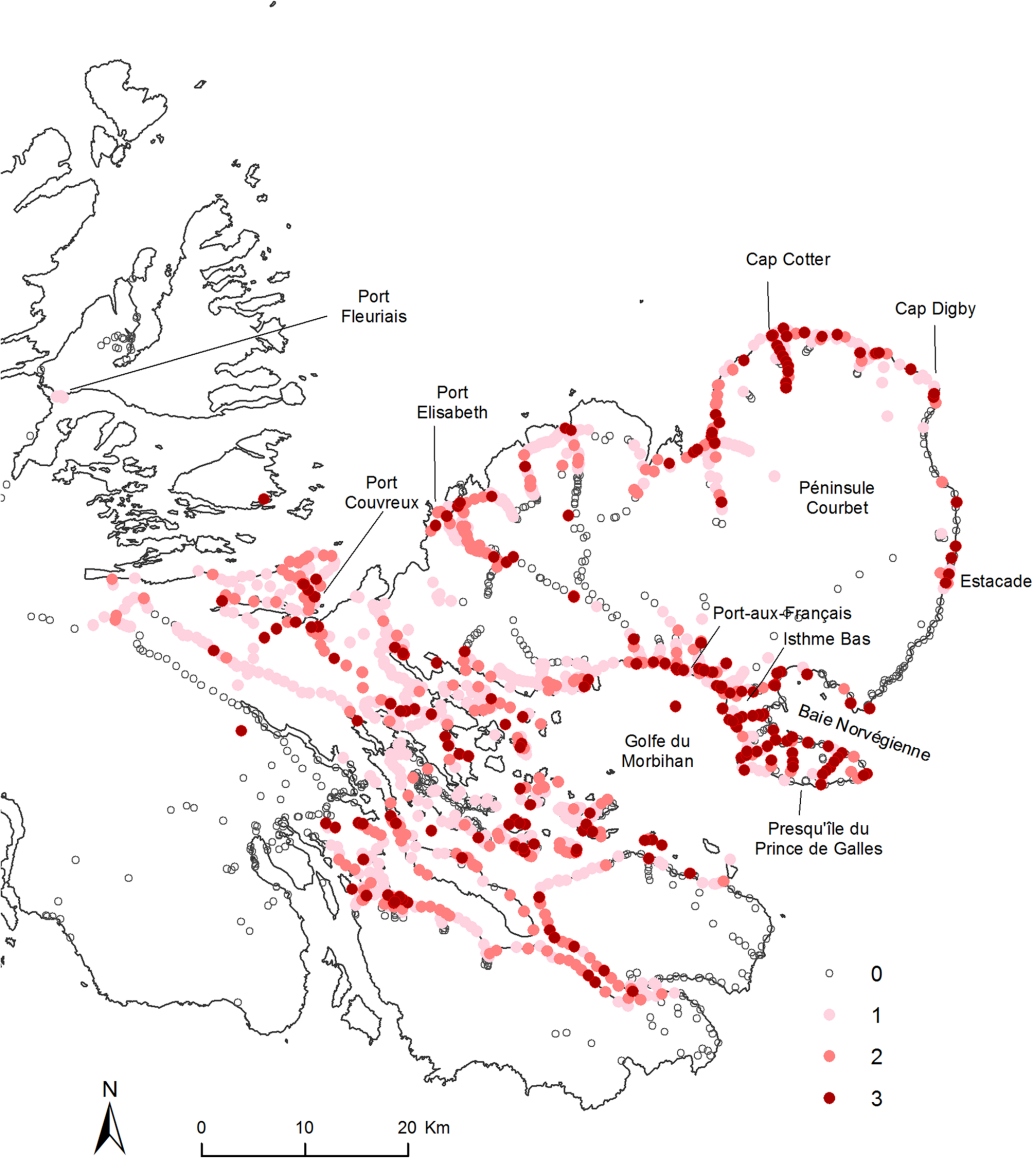
Map of all observations reporting the distribution of *Merizodus soledadinus* on the Kerguelen Islands between 1939 and 2018. Each observation is plotted according to the number of adults found during a 10-min search: (0) absence, (1) low abundance, 1–30 adults, (2) medium abundance, 31–100 adults, and (3) high abundance, more than 100 adults. The map of the Kerguelen Islands was created by the authors; the background map we used for the figure is not a copyrightable subject matter. Geospatial data were incorporated into the map with ArcMap in ArcGis 10.4 (https://www.esri.com), and this software was used to generate the map.

By 2018 (Fig. 3) the beetle’s distribution had expanded considerably in the interior of the Péninsule Courbet, in the valley between Port-aux-Français and Port-Elizabeth. On the east coast, the entire Baie Norvégienne was colonised, as were several locations between Estacade and Cap Digby. In the Golfe du Morbihan, the species has now been recorded from all surveyed islands and islets. However, to date, it has not been observed in the western part of the archipelago (Massif Gallieni, Péninsule Loranchet and Péninsule Rallier du Baty).

**Figure 4:**
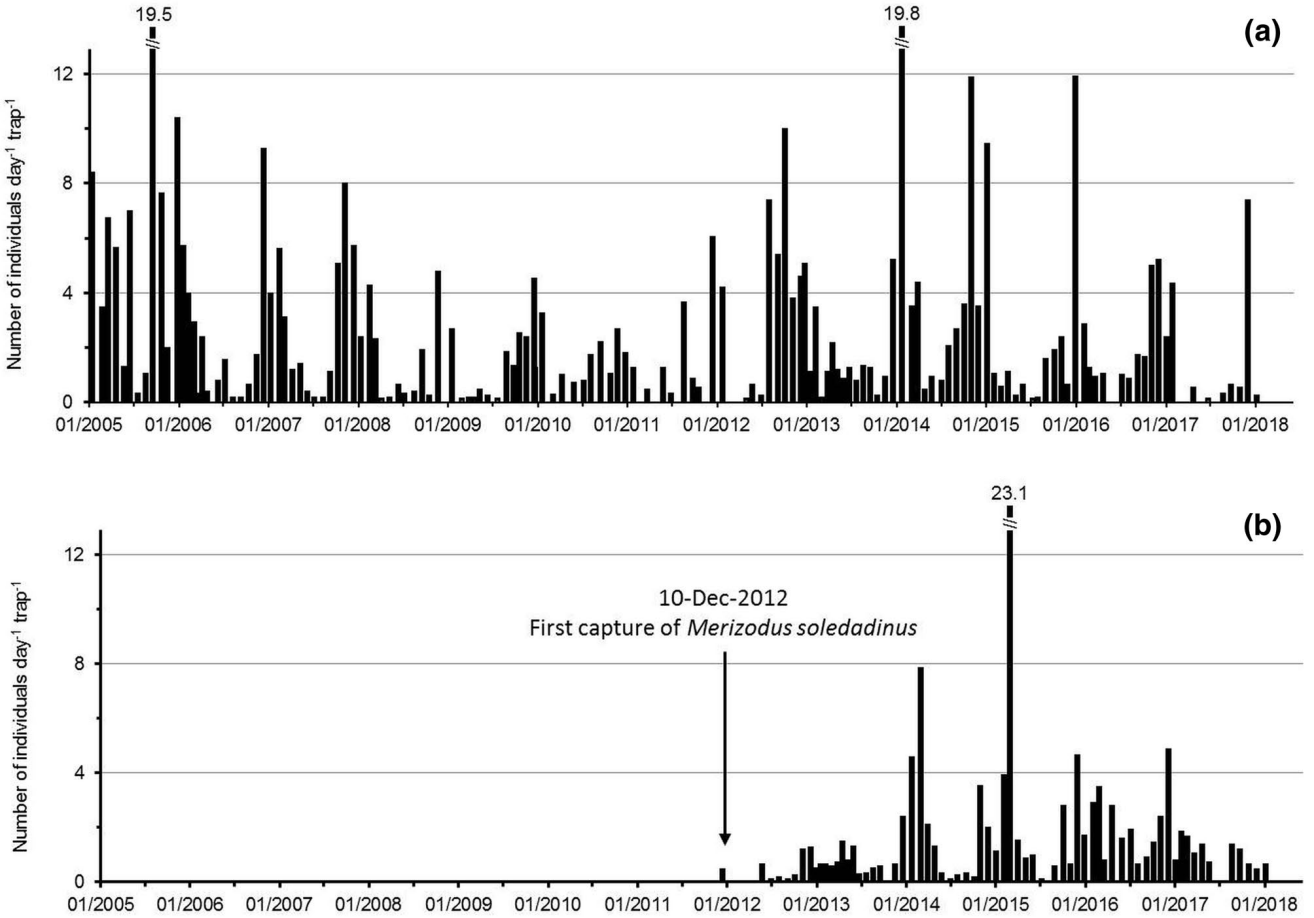
Captures of *Merizodus soledadinus* at two sites on the Kerguelen Islands between 2005 and 2018: (**a**) west coast of Isthme-Bas, site colonized between 2000 and 2005, (**b**) east coast of Isthme-Bas, first observation at this site in 2012.

An abundance index was applied to each of the 1164 locations (Fig. 3) where the species was recorded between 2005 and 2018 (after a 10 min active search following a standard protocol: low abundance 1–30 adults, n = 680 observations; medium abundance 31–100 adults, n = 265 observations; high abundance > 100 adults, n = 219 observations). To illustrate an anecdotal impression of the highest abundance category, in one case it took less than 3 min to find 150 individuals under a single stone of c. 200 cm^2^ area. The beetle was particularly abundant in the vicinity of Port-aux-Français, on Presqu’île du Prince de Galles, and between Cap Cotter and Cap Digby, with more than 300 individuals being routinely counted in 10 min in 2013. It is notable that instances of high abundance were found throughout the colonized area, on the coast, on the islands of the Golfe du Morbihan, and inland, in both recent and older colonized habitats, suggesting that its population density can increase quickly wherever it becomes established. This suggestion is also supported by monthly trapping data initiated in 2005 on both sides of Isthme Bas, where the number of adults captured on the east coast, colonized in 2011, rapidly reached similar levels to those recorded from the west coast, colonized between 2000 and 2005 (Fig. 4).

### Ecological impact of *Merizodus soledadinus* on the native dipterans *Anatalanta aptera* and *Calycopteryx moseleyi*

**Figure 5:**
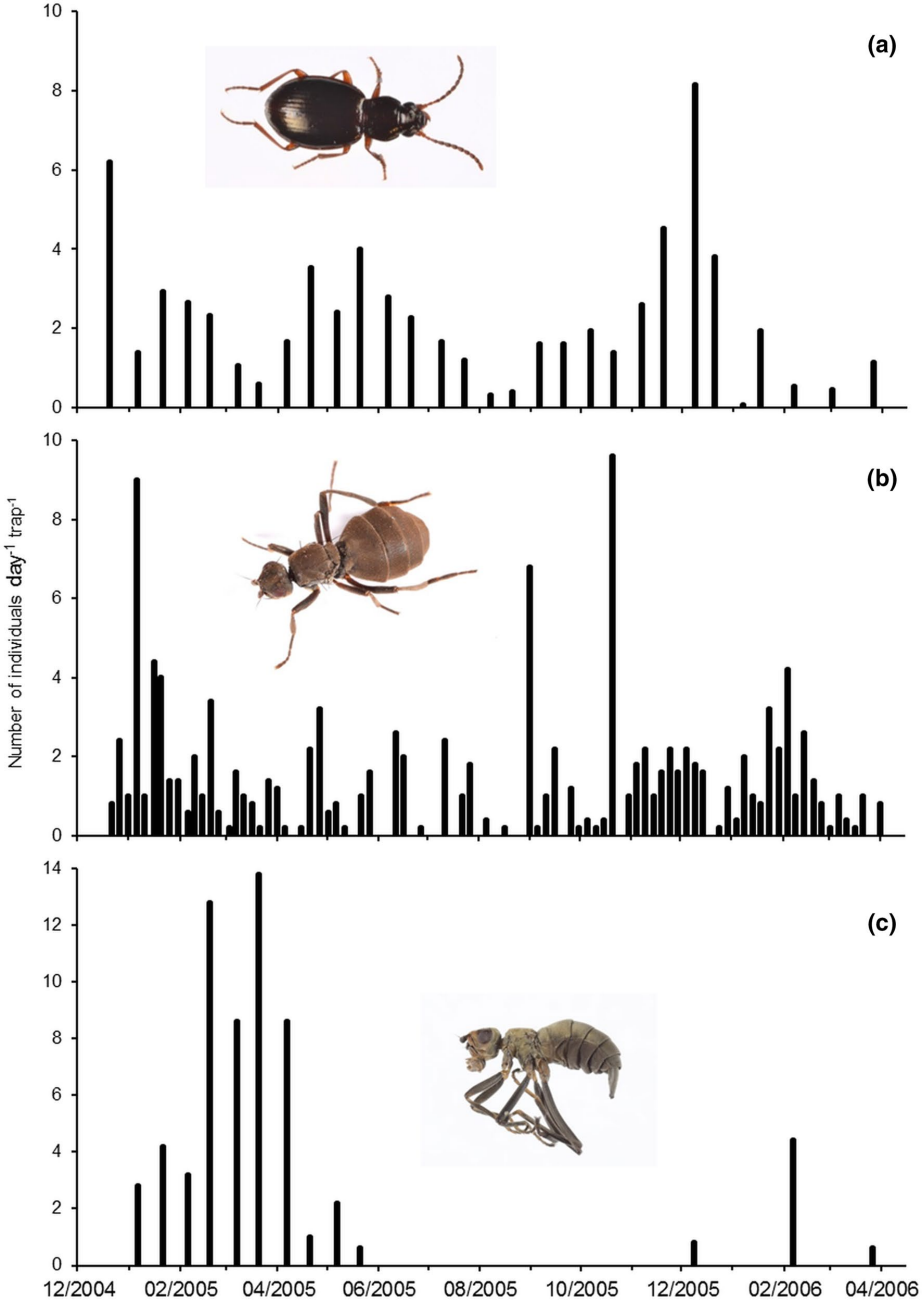
Patterns of activity on the Kerguelen Islands of (**a**) *Merizodus soledadinus*, (**b**) *Anatalanta aptera* and (**c**) *Calycopteryx moseleyi* as indicated by trapping conducted at Port-aux-Français. Photo credit: Project IPEV 136 Subanteco (PI: D. Renault).

We considered abundance data for these three insects. Between December 2004 and March 2006, populations of *M. soledadinus* and *A. aptera* fluctuated seasonally, and declined during winter (Fig. 5a,b), with less than 10% of the records obtained (total records = 862) being during the austral winter in July and August. For *A. aptera*, expected frequencies significantly differed from observed frequencies (Table 1) along the seashore (n = 338, χ^2^ = 38.877, *p* < 0.001), inland (n = 379, χ^2^ = 54.217, *p* < 0.001) or under carrion (n = 155, χ^2^ = 15.560, *p* < 0.001): *A. aptera* was more often present and abundant than expected when *M. soledadinus* was absent; conversely, the fly was more often than expected absent or at low abundance when *M. soledadinus* was present.

**Table 1:** Frequency distribution of the abundance of adult *Anatalanta aptera* (Aa) and *Merizodus soledadinus* (Ms) in three habitats (seashore, inland, carrion).

Seashore			Abundance of *M. soledadinus*				
			0	1	2	3	Total Obs
**Abundance of** ***A. aptera***	0	Observed	1	5	53	32	91
		Expected	37.5	42.3	21.9	6.7	
	1	Observed	66	59	30	10	165
		Expected	56.7	36.6	33.5	10.2	
	2	Observed	29	14	3	2	48
		Expected	16.5	18.8	9.7	3.0	
	3	Observed	12	13	7	2	34
		Expected	11.7	13.5	6.9	2.1	
	Total Obs		108	91	93	46	338

Inland			Occurrence of *M. soledadinus*				
			0	1	2–3		Total Obs
**Occurrence of** ***A. aptera***	Aa absent	Observed	83	129	35		247
		Expected	116.0	106.9	24.1		
	Aa present	Observed	95	35	2		132
		Expected	61.9	57.1	12.9		
	Total Obs		178	164	37		379

Under carrion			Abundance of *M. soledadinus*				
			Ms absent	Ms present			Total Obs
**Abundance of** ***A. aptera***	0	Observed	7	17			24
		Expected	15.5	8.5			
	1	Observed	87	36			123
		Expected	79.3	43.6			
	2–3	Observed	6	2			8
		Expected	5.2	2.8			
	Total Obs		100	155			155

Codes for abundance according to the number of adults found during a 10 min active search: 0 = absent; 1 = low abundance, 1–30 adults; 2 = medium abundance, 31–100 adults; 3 = high abundance, > 100 adults. When information on the abundance of the insects was not available, occurrence is reported as absence/presence only. Expected frequencies in italics. Observed frequencies significantly differed from expected frequencies along the seashore (χ^2^ = 38.877, *p* < 0.001), inland (χ^2^ = 54.217, *p* < 0.001) and under carrion (χ^2^ = 15.560, *p* < 0.001).

As adults of *C. moseleyi* were not recorded throughout the whole year (Fig. 5c), we considered the records of *M. soledadinus* and *C. moseleyi* only from the highest abundance period of *C. moseleyi*, i.e. from January to March 2005 (n = 177). Here again, expected frequencies significantly differed from observed frequencies (Table 2) (χ^2^ = 36.007, *p* < 0.001). Numbers of *M. soledadinus* negatively affected the abundance of adult *C. moseleyi*: when the ground beetle was present within the habitat, *C. moseleyi* was more often absent (0) and less often abundant (abundance index 2 or 3) than predicted.

**Table 2:** Frequency distribution of the abundance of adult *Calycopteryx moseleyi* (Cm) and *Merizodus soledadinus* (Ms) along the seashore.

			Occurrence of *M. soledadinus*		
			Absent	Present	Total Obs
**Abundance of** ***C. moseleyi***	0	Observed	15	97	112
		Expected	31.5	73.5	
	1	Observed	30	26	56
		Expected	16.8	39.2	
	2 and 3	Observed	6	3	9
		Expected	2.7	6.3	
	Total Obs		51	126	177

Codes for abundance according to the number of adults found during a 10 min active search: 0 = absent; 1 = low abundance, 1–30 adults; 2 = medium abundance, 31–100 adults; 3 = high abundance, > 100 adults. As information on the abundance of *M. soledadinus* was not always available, occurrence is reported as absence/presence only. Observed frequencies significantly differed from expected frequencies along the seashore (χ^2^ = 36.007, *p* < 0.001).

**Figure 6:**
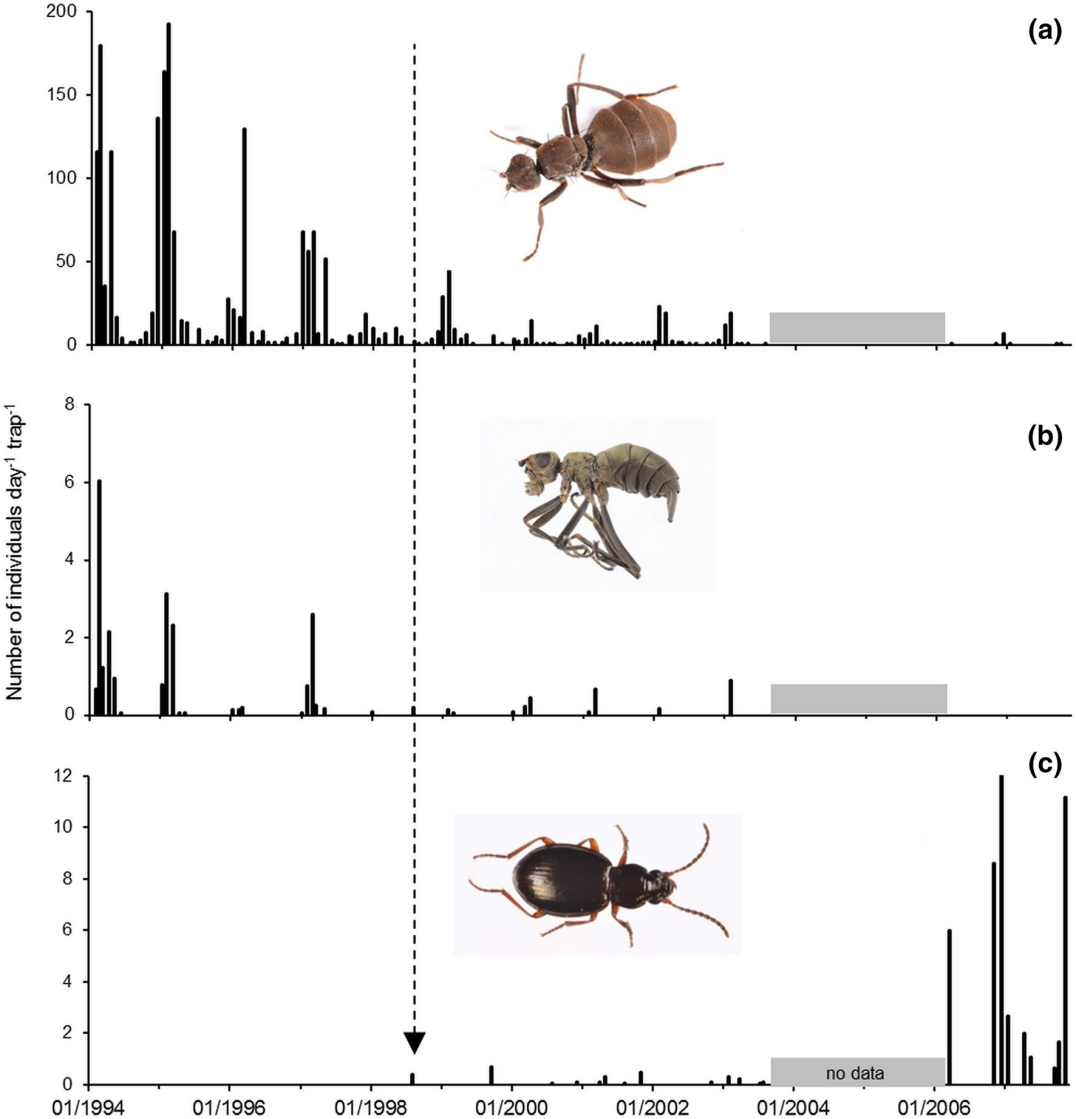
Captures of invertebrates by trapping at Île Guillou, Kerguelen Islands, between 1994 and 2007. The arrow indicates the date (30-Jul-1998) of the first record of *M. soledadinus* at this site. (**a**) *Anatalanta aptera*, (**b**) *Calycopteryx moseleyi*, and (**c**) *Merizodus soledadinus*. Photo credit: Project IPEV 136 Subanteco (PI: D. Renault).

Additional information on the impact of *M. soledadinus* on *A. aptera* and *C. moseleyi* was provided by trapping results from a coastal site on Île Guillou. Trap records of the two native flies were consistent over the austral summers 1994–1997, before the establishment of the beetle at this location, with lower but still consistent numbers of adult *C. moseleyi* (1–3 per trap per day). When *M. soledadinus* was first trapped in July 1998 at this site, both flies were still present. The beetle was then regularly trapped until 2003 (0.1–0.7 individuals per trap per day in 13 of the 54 trapping sessions). Over this period a drastic decrease in records of *A. aptera* and *C. moseleyi* became apparent (Fig. 6). After a pause in trapping, when it was resumed in 2006, *M. soledadinus* was recorded in every trapping session and at higher abundance than previously (1–11 individuals per trap per day, with a maximum value of 46 in December 2006). However, the numbers of *A. aptera* caught were extremely low, and *C. moseleyi* was no longer recorded.

## Discussion

The geographical expansion of distributions of non-native species is a key topic in invasion science and has considerable significance to the management of invading populations^69^. Human-assisted introduction and spread of non-native organisms typically involves multiple introduction points, and the large spatial scales involved often impede in-field monitoring of the distribution and abundance of the species involved^70^. In the present study, we were able to take advantage of a more tractable invasion study system, that of the accidentally introduced carabid beetle *M. soledadinus* on the Kerguelen Islands archipelago, which allowed us to report a combination of spatial and temporal in-field monitoring of the processes of geographical distribution expansion of this non-native insect. We additionally highlight how quickly the population of this insect builds up when establishing in a new habitat, and how much the geographic expansion of this invasive predator could contribute to the depletion of native insect communities in this UNESCO World Heritage Area.

### Historical documentation of the invasion

In February 1939 when *M. soledadinus* was first observed, already locally abundant, on the Kerguelen Islands^37^, it was restricted to the surroundings of the abandoned farm buildings of Port-Couvreux, suggesting a recent introduction^37^. Jeannel^37^ first hypothesized that the activities of American sealers provided the probable source of the introduction in the archipelago, in particular the ship ‘*Hillsborough*’ which landed at Port-Couvreux in 1799. However, he later revised this assumption, taking the view that the species would have had a considerably larger distribution range on the archipelago if it had been introduced around 1800^44^. Hence, he suggested that individuals could have been introduced at Port-Couvreux when the farm buildings (piggery, sheepfold) of this locality were enlarged in 1927–1928^44,68^.

For the purpose of this study, we identified the very limited number of vessels recorded to have landed at Port-Couvreux, and their previous itinerary (including landings in other harbours / regions)^67^ before the first specimens of *M. soledadinus* were observed. Taking into account the narrow austral native distribution of the species (southern South America, Falkland Islands)^41,46,62–64^, we conclude that Jeannel’s revised introduction assumption also cannot be supported. The sheep farming attempt that took place in 1927 used animals loaded during a call at Durban (South Africa) by the ship ‘Lozère’, which was transporting material originally from Le Havre (France). This vessel did not visit anywhere in the region of the beetle’s natural distribution. Based on our review of the available shipping records, we identified the ‘*Jacques*’, a vessel belonging to René Bossière who, with his brother, established the farm of Port Couvreux, as the most likely introduction source^67^. The ship left Swansea (United Kingdom) in February 1913, and sailed via Montevideo (Uruguay; not within the natural distribution of *M. soledadinus*) to the Falkland Islands^67^ where the species is native^46^. Here, the vessel remained about 1 month and loaded equipment and ca. 1600 sheep^67^. The tussock grass *Poa flabellata* Raspail, 1829 (Poaceae) is common in coastal areas of the Falkland Islands^45^, and provides a habitat for *M. soledadinus*^51^. As this grass can be used as a fodder source, it is very likely that fodder harvested in the Falkland Islands to feed sheep during their transport to the Kerguelen Islands contained the insect. In August 1913, the ‘*Jacques*’ arrived at Port-Couvreux where the 1150 surviving sheep and the remaining fodder were unloaded^67^. Additional circumstantial support for a single introduction event is the presence at Port-Couvreux (and, to date, nowhere else on the Kerguelen Islands) of *Trisetum spicatum* (L.) K.Richt., 1890 (Poaceae); this grass, which is widespread in cold regions of both the Northern and Southern Hemispheres, but not on the islands of the southern Indian Ocean sub-Antarctic province, is also present on the Falkland Islands^71^. No other vessels are recorded as sailing from regions where *M. soledadinus* was distributed at that time and landing at Port-Couvreux in the period between 1893—when the Bossière brothers obtained the concession from the French government to exploit the resources of the Kerguelen Islands—and 1939—when *M. soledadinus* was first observed by Jeannel^67^. Taken together, these historical records suggest the landing of the ‘*Jacques*’ at Port-Couvreux in 1913 as the most likely introduction scenario of the beetle at the Kerguelen Islands.

### Temporal and spatial spread

After its initial introduction at Port-Couvreux in 1913, the species persisted without expanding its range, at least until 1939, when Jeannel^37^ found it at high density at the introduction site but also actively searched for and failed to find the species at multiple sites in the archipelago. A lag phase is commonly reported in studies of invasion processes, with its duration varying across taxa and with the characteristics of the introduction sites. For instance, Kiritani and Yamamura^72^ reported a mean lag phase of 11.8 years for the 35 non-native insects they considered, in line with the predicted lag range of 4.4–23.2 years of Morimoto et al.^73^. Similarly, the gypsy moth *Lymantria dispar* took about 20 years to spread only 500 m from its initial introduction point in the USA^74^, before its subsequent rapid expansion commenced. Thus, even if recent models suggest that it is not possible to make accurate predictions of the duration of lag phases, in particular for introductions occurring in coastal areas^19^ as in the case for *M. soledadinus*, the apparent lag time for this species is consistent with the existing insect invasion literature. The lag time could in part result from Allee effects, which refer to any process whereby any component of individual fitness is correlated with population size (^76,77^, also see the reviews of^6,78,79^, which describe the different concepts underlying invasion dynamics). Investigations conducted on populations of *M. soledadinus* support this idea. For instance, while adult beetles have a relatively long lifespan of 1–2 years^79^, the small (3–12) mature egg load per female, and the long developmental period of the juveniles (at least several months^55,61,79^), may limit individual opportunities to find a mate. Such life history characteristics may have contributed to restricting population growth in the initial years following the species’ introduction at Port-Couvreux. Inbreeding depression is a further potential element of the Allee effect^76^, although there is no direct evidence of it playing a role in the establishment of *M. soledadinus* in the archipelago. However, preliminary studies have found that adults of *M. soledadinus* obtained from Port-Couvreux exhibited significantly lower levels of heterozygosity than those from native Patagonian populations^80^.

Farming and the human presence at Port-Couvreux ceased in 1931^67^, after which there was little human presence or activity on the Kerguelen Islands until the early 1950s, when the scientific research station Port-aux-Français was established. Thus, initial expansion in distribution of the beetle from Port-Couvreux (Presqu'Île Bouquet de la Grye) did not occur with human assistance, at least until it reached Port-aux-Français (Péninsule Courbet) in the late 1990s. During the austral summer of 1982–1983, confirmation of its presence in the general vicinity of its introduction site (Fig. 2) is consistent with natural dispersal. As well as terrestrial dispersal, some beetles may have directly crossed the inlet separating Presqu'Île Bouquet de la Grye and Plateau Central (Fig. 2) by marine rafting. While the distance from Port-Couvreux to Anse Sablonneuse is 25 km overland, it is only 400 m by direct line crossing the inlet, and experimental data have revealed that *M. soledadinus* can survive flotation and exposure to saline conditions for several days^81,82^.

Habitat connectedness is a key influence on dispersal performance in insects^83,84^. In particular, a lower landscape permeability in between two patches can restrict geographic expansion in insects^85^. In this context, large rivers or areas of non-vegetated coastline appeared to have acted at least temporarily as local geographical barriers to the spread of *M. soledadinus*. Barrier zones can be very efficient in limiting the expansion of invasive insects, as modelled in the gypsy moth^86^. Here, we found that the presence of steep cliffs in the south of Péninsule du Prince de Galles seemed to halt the expansion of the beetle. Earlier studies elsewhere also reported that geographic barriers, while larger than those suggested here, can prevent the dispersal of invasive insects. Such examples include the Colorado potato and mountain pine beetles, whose spread may have been limited by geoclimatic conditions and the Rocky Mountains^87,88^. In line with other investigations^89^, there is a possibility that local biodiversity may provide a significant barrier to the spread of non-native organisms on Kerguelen. For instance, at Cap Digby, the presence of a large penguin colony with probable effects on soil composition^90^ may limit movement of *M. soledadinus* along the coast.

While roads and rivers are well-known dispersal corridors accelerating the geographic expansion of invasive species^91–93^, here we conclude that the seashore is the most prominent dispersal corridor for *M. soledadinus* in the Kerguelen archipelago, providing connectivity between areas of habitat. Physiological studies have reported that humidity and water availability are key factors that can quickly impair the survival of adults^94^. However, ponds, waterlogged areas, streams and rivers are frequent in habitats close to the seashore around the archipelago, often combined with the presence of abundant food resources in the form of native and other non-native insects^33,36,95^. Temperature likely represents an additional factor driving the expansion of this species, whose spread rate of ca. 3.0 km/year (Île Haute)^57^ is far higher than that estimated on the colder South Georgia (0.1 km/year^61^) where it has also been introduced. However, at the time of the latter study in the 1980s, the species may have still been in the lag phase. Although our data do not allow formal spread rate calculations, assuming that the flightless *M. soledadinus* invaded the south coast of Péninsule Courbet from the research station of Port-aux-Français from 1999 onwards, and based on our in-field surveys, the spread rate achieved would be between 1.7 and 2.7 km/year. The spread rate of *M. soledadinus* at the Kerguelen Islands is thus similar to that of closely related carabid beetles, such as *Trechus obtusus* Erichson, 1837 (3.0 km/year in Hawaii^96,97^).

The arrival of *M. soledadinus* in the vicinity of the research station at Port-aux-Français (first observed in 1999) marked a significant milestone in the beetle’s expansion in the archipelago. Its presence in an area of considerable human activity created the opportunity for further human assistance in dispersal within the archipelago. A clear example of this is given by Estacade, a location with a field hut that has long been used to support monitoring of penguin populations and, to a lesser extent, by tourists who previously were able to overnight when visiting the penguin colonies. When the beetle was first observed at Estacade in 2005 (after most probable establishment between 1995 and 2000, as the shelter was not used frequently after early 2000), the closest established populations of *M. soledadinus* were at Cap Digby and Port-aux-Français, both more than 20 km distant from Estacade. It is thus very likely that accidental human transport of small numbers of beetles was responsible for their introduction to Estacade.

However, even with the likelihood that human assistance has played a role in further spreading the beetle after its arrival in the vicinity of the research station, this is unlikely to explain all instances of local colonisation by the beetle, for instance of several islands of the Golfe du Morbihan that are rarely visited. Rather, as suggested earlier, beetles are likely to have arrived by rafting on vegetation or algae^81,82^ or through ornithochory. For instance, Kerguelen shags *Phalacrocorax verrucosus* use seaweeds that they sometimes transport from one island to another when building their nests. Consistent with this, carrion and skulls of rabbits (*Oryctolagus cuniculus*) are regularly found on the north-west coast of Île Australia, an island where rabbits do not occur. Carrion typically hosts *M. soledadinus* individuals which prey on fly larvae developing on the cadavers^38^. The transport and consumption of carrion by scavenging birds (e.g. skuas *Stercorarius antarcticus lonnbergi* and giant petrels *Macronestes giganteus*) thus represents another possible mechanism of dispersal for the beetle.

Abundances of *M. soledadinus* recorded in this study are considerably greater than those reported in the early 1990s^57^. They are also much higher than those reported from South Georgia (maximum = 156 collected per hour^61^). Even though individuals of *M. soledadinus* have well developed thermal stress tolerance^98^, and have colonized low altitudes on South Georgia^99^, the harsher climatic conditions of South Georgia as compared with the Kerguelen Islands may reduce the beetle’s abundance and the speed of its geographic expansion. Mean annual temperature on King Edward Point during the period 1951–1980 was 2.0 °C and snow cover was more or less permanent from May to October in the coastal area^55^. During the same period (1951–1980), the mean annual temperature was 4.5 °C at Port-aux-Français, and despite regular snowfall (ca. 15 days per month from June to August), no permanent snow cover was observed (Source: Meteo France data).

Earlier studies assessing the physiological capabilities of *M. soledadinus* reported limited signs of thermal stress in adults permanently exposed to temperatures as high as 20 °C^99,100^, and suggested that warming may further assist the local spread of the species. In the Kerguelen Islands, air temperature increases that occurred during the winter months in the early 1990s resulted in a 20–30 days’ reduction of the number of freezing days each year (Source: Meteo France data^33^). Warming may represent a significant driver of the recent colonization of moderate altitudes by populations of *M. soledadinus*. The highest altitude that established *M. soledadinus* was recorded was 110 m above the sea level (asl) in the mid-1990s^36^, while populations were found up to 358 m asl in 2005 (this study,^101^). Comparing the morphological and biochemical characteristics, and metabolic phenotypes, of adult *M. soledadinus* sampled along altitudinal transects, Ouisse et al.^101^ concluded that the presence of the insects at moderate altitudes resulted from the progressively higher occurrence of thermally suitable habitats.

### Ecological impacts

Alien insects can severely affect native biodiversity, in particular when they are predators bringing novel ecological function into the invaded habitats and are no longer limited by other predators. As insects, they can also often develop large population densities, further increasing the impacts they can have on prey species. The invasion process of the predaceous *M. soledadinus* has had major impacts on the native entomofauna, even in the most recently colonized locations, where *M. soledadinus* rapidly becomes dominant. The native flies *A. aptera* and *C. moseleyi* appear to have been largely lost or even driven locally extinct in several locations colonized by *M. soledadinus* (^33,39^, this study). We also highlight that *C. moseleyi*, whose population densities are often much lower than those of *A. aptera,* is more sensitive to the year-round active *M. soledadinus*^79^ than *A. aptera* in seashore habitats. This is particularly critical for the endemic *C. moseleyi*, as algae already represent its secondary trophic niche after its primary resource (the Kerguelen cabbage, *Pringlea antiscorbutica*^95^) was wiped out from most locations invaded by rabbits^102^.

On Île Guillou, the number of *A. aptera* caught dropped from about 50–10 individuals per day per trap during the austral summer 1998, and almost no *C. moseleyi* were found in the traps. On Île Guillou, the low seasonality in the life cycle of *M. soledadinus*, the September to April emergence period peaking in February–March^79^, and the several months necessary for the development of juvenile sub-Antarctic carabids^55,56^ suggest an introduction of a small number of *M. soledadinus* adults during the austral summer of 1997. The subsequent establishment, reproduction and development of the carnivorous larvae and adults of the insect are consistent with the reported declines in numbers of native flies in the following austral summer (e.g., 1998), a few weeks before the first collection of adults. The surveys conducted at Isthme Bas support this assumption, where trapping reveals that it takes around two consecutive austral summers before the number of collected adult *M. soledadinus* exceeds 1.5 insect per trap per day on average. Interactions of *M. soledadinus* are likely to occur with the three native arthropod predators, the rove beetle *Leptusa atriceps* (Waterhouse, 1875) (Coleoptera: Staphylinidae) and the linyphiid spiders *Myro kerguelensis* (Pickard-Cambridge, 1876) (Araneae, Desidae) and *Neomaso antarcticus* (Hickman, 1939) (Araneae: Linyphiidae). Direct predation may occur, with late-instar larvae and adults of *M. soledadinus* predating small spiders. Adult *M. kerguelensis* may be capable of predating larvae of the ground beetle. In South Georgia 217 *Trechisibus antarcticus* (Dejean, 1831) (Coleoptera: Carabidae) and 68 spiders were found in one litter sample, all of the latter much smaller than *M. soledadinus*^55^. Spiders may prey on springtails and be preyed upon by the carabid^55^. At South Georgia, *M. soledadinus* also has a strong impact on the abundance of the endemic perimylopid beetle *Hydromedion sparsutum* (Waterhouse, 1875) (Coleoptera: Perimylopidae)^65^. Taken together, our data and the available literature suggest that *M. soledadinus* can have profound impacts on native insect species, acting as an ecosystem engineer and changing significantly the invaded habitats along the invasion gradient.

## Conclusions

Although much less visible than the rabbit, which has completely altered vegetation structure and cover in the areas it has colonized in the Kerguelen Islands^103^, the impact of *M. soledadinus* on the archipelago’s terrestrial ecosystems is considerable. The wide-reaching consequences on biodiversity produced by this predatory species create novel disturbances in the Kerguelen Islands, where the beetle is an invasive ecosystem engineer. Despite its inability to fly, its distribution in the archipelago is continuing to expand. This has been especially apparent since its arrival in the vicinity of the active research station, from where inadvertent human assistance has accelerated its geographic spread. No control measures, let alone eradication, are practicable, so it is urgent and essential to limit as far as possible any further dispersal by human activities. Our study confirms the crucial importance of long-term biosurveillance for the detection and subsequent monitoring of non-native species and the timely implementation of control measures. With the species already having a wide distribution in parts of the archipelago, knowledge of its past and current distribution provide valuable insight into the environmental drivers of its geographic spread, and help identify suitable habitats vulnerable to colonisation by the species. This body of knowledge, which could serve for making predictions on future geographic expansion^104^, underpins the current urgent requirement to define and implement effective biosecurity measures (i.e. detailed inspection and cleaning of clothing, footwear, equipment, freight, vehicles and vessels) in concert with the management of the National Nature Reserve, in particular when visiting sites not yet colonized by *M. soledadinus*. More widely, similar priority must be given to minimising the risk of transfer to other ‘at risk’ sub-Antarctic and lower latitude islands, in particular the Crozet Islands, Amsterdam and St. Paul Island, and La Reunion, all of which are served by both the French sub-Antarctic logistic operation, National Nature Reserve, and ships of the tourist industry. These data can also be used in the development of predictive species distribution models under different climate change and management scenarios, further supporting the optimization of future management strategies.

## Materials and methods

### Study area

The study was conducted on the Kerguelen Islands (48° 30′–50° S, 68° 27′–70° 35′ E), a sub-Antarctic archipelago located in the Southern Indian Ocean more than 3500 km from the African and Australian coasts. This archipelago (total area 7200 km^2^) consists of a main island (6500 km^2^), over 10 smaller islands (100–200 km^2^), and numerous islets (> 1 km^2^). The highest point is Mont Ross (1850 m), and an ice cap (Glacier Cook) is present in the western sector (Fig. 1). There is no permanent population, but the research station (Port-aux-Français) established in 1950 hosts 50–100 persons year-round. Gravel roads are restricted to the vicinity of the research station, and human activities, i.e. research, logistics, and a limited amount of tourism, mainly take place in the eastern sector of the archipelago (Péninsule Courbet, Golfe du Morbihan, Péninsule Jeanne d'Arc). Visits to more remote sites are limited and involve the use of vessels or helicopters that are not permanently available on the island. In 2006, the Kerguelen Islands were given the status of national nature reserve, the highest level of protection available under French law, and some wilderness areas were classified as “strict nature reserve” where human access, use, and impacts are strictly controlled and limited. Given the outstanding value of the archipelago, it has recently been designated as a UNESCO World Heritage Area.

### Biological models

*Merizodus soledadinus* (Guérin-Méneville, 1830) (Coleoptera: Carabidae) is a flightless carabid beetle naturally distributed in southern South America (Patagonia) and the Falkland Islands^41,46,105–107^. It was first described in 1830 (as *Trechus soledadinus*) by Guérin-Méneville, from the Falkland Islands (Soledad Bay). Later, Enderlein^107^ also reported the insect from the Falkland Islands as *Dormeyeria soledadina*. In 1940, Jeannel named the species *M. soledadinus,* with subsequent renaming to *Oopterus soledadinus* by Johns^108^. Lalouette^109^ restored *M. soledadinus*, further confirmed by Voisin et al.^110^. *Merizodus soledadinus* has been accidentally introduced to two sub-Antarctic islands or archipelagos, the Kerguelen Islands (first record in 1939^37^) and South Georgia (first record in 1963^47^). On Kerguelen, adults have been described as being active at night^53^ and are found during the day beneath stones and kelp belts^55^.

*Anatalanta aptera* Eaton 1875 (Diptera: Sphaeroceridae) is a wingless fly that is endemic to the Indian Ocean Province sub-Antarctic islands. It can be found on the Crozet and Kerguelen archipelagos and on Heard and McDonald Islands. On the Kerguelen Islands, it is present from sea level to more than 600 m asl and is active year-round. Larvae and adults are saprophagous and feed on decaying organic matter. *Anatalanta aptera* is abundant in many habitats, especially in seabird colonies, around carrion and in coastal areas enriched by seaweeds^39,111^.

*Calycopteryx moseleyi* Eaton 1875 (Diptera: Micropezidae) is another wingless fly endemic to the Indian Ocean Province sub-Antarctic islands. It can be found in the Kerguelen archipelago and on Heard and McDonald Islands. Its larvae feed preferentially on the Kerguelen cabbage *Pringlea antiscorbutica*, but can also frequently be found under decomposing seaweeds along the seashore and on decaying organic matter in penguin rookeries^94^.

### Historical documentation of the presence of *Merizodus soledadinus* on the Kerguelen Islands

Within the existing literature, suggested introduction dates of *M. soledadinus* to the Kerguelen Islands vary between 1800^37^, 1913^39^ or 1927^43^. In a search for literature aimed at clarifying the species’ introduction history, we used search strings in Web of Science encompassing the terms ‘Kerguelen Islands’, ‘Kerguelen archipelago’, ‘Iles Kerguelen’, ‘*Oopterus*’, ‘*soledadinus*’, ‘*Merizodus*’, ‘vessel’, ‘ship’, ‘sheep’, ‘Bossière’, ‘Port-Couvreux’, ‘Falkland Islands’, ‘Patagonia’, ‘Chile’, ‘Argentina’, and ‘South Georgia’. The results obtained were then manually checked to retain only those that were relevant to our historical documentation. The cited literature of the selected documents was also examined. The literature search was further complemented with (1) information published by our group since 2010, and (2) observations of the distribution of *M. soledadinus* recorded since the 1990s within the framework of the long-term sub-Antarctic programme IPEV 136. Three books describing the history of the Kerguelen Islands^66–68^, including an exhaustive list of the vessels that landed in the archipelago before the construction of the research station in the early 1950s, were also consulted to address the objectives of this study. The combination of literature search and more recent data provided valuable information pertinent to the introduction history and expansion of *M. soledadinus* in the archipelago.

### Long-term monitoring of the invasion of the Kerguelen Islands by *Merizodus soledadinus*

To assess changes in the geographic distribution of the beetle, we first analyzed more than 2000 presence/absence records of *M. soledadinus* collected over the 1991–2018 period. From 1991 to 2005, georeferenced distribution occurrences of *M. soledadinus* were recorded as a part of long-term monitoring of the biodiversity of the Kerguelen Islands (Programme IPEV 136 Subanteco). In addition the presence/absence of the species was noted opportunistically during visits to other localities.

From December 2004 to March 2006, a systematic exhaustive survey of the geographical distribution and abundance of *M. soledadinus* was conducted in the archipelago. This initially focussed along the coastline where the species had long remained confined^39^. At each site surveyed, we noted the GPS coordinates of the record, the occurrence (presence/absence) and abundance (abundance index) of *M. soledadinus*, in addition to recording the occurrence of the two native flies *A. aptera* and *C. moseleyi*. For the abundance index, preliminary trials showed that a 10-min search by one person was appropriate to detect the presence of the three insects and make accurate estimates of their densities, even when they were present in low numbers. Thus, a semi-quantitative index was designed based on the number of adults found during the 10-min search: (0) absence, (1) low abundance, 1–30 adults, (2) medium abundance, 31–100 adults, and (3) high abundance, more than 100 adults. The nature of microhabitats hosting the three insects was also recorded, and resulted in the following list: stranded seaweed, stones, carrion, and leaves of the Kerguelen cabbage. Inland sites were subsequently surveyed by making the same observations along transects moving inland perpendicular to the shoreline and along altitudinal transects. Point surveys were completed ca. every 100 m along the former and ca. every 20 m in elevation along the latter in order to accurately define *M. soledadinus* distribution limits at the edges of colonized areas. Observations were stopped when no *M. soledadinus* were observed at two consecutive survey sites.

Finally, since 2006, as a part of systematic recording of the distribution of entomofauna at the Kerguelen Islands, further georeferenced field surveys of *M. soledadinus* have been carried out, paying particular attention to conducting active searches at range edges of the species’ known distribution.

### Assessment of population dynamics and seasonal fluctuations of *Merizodus soledadinus* in colonized habitats

To clarify the beetle’s population dynamics after its establishment in a new habitat, we monitored invertebrate communities at two sites on Péninsule Courbet from 2005 to 2018. For this long-term monitoring study, three pitfall traps (Ø 9 cm, h 4 cm) were opened for 5 d every 2–3 weeks at each sampling site. In the first site, on the west coast of Isthme Bas, *M. soledadinus* established between 2000 and 2005. At the second site, on the east coast of Isthme Bas, *M. soledadinus* was absent at the start of the study, and became established during the monitoring period. At both sites, the pitfalls were placed in herbfield communities dominated by the deciduous dwarf shrub *Acaena magellanica* (Vahl 1804) (Equisetopsidae: Rosales). After collection, beetles were stored in 70% ethanol, identified to species level, and counted in the laboratory.

### Ecological impact of *Merizodus soledadinus* on native entomofauna

To assess the ecological impact of *M. soledadinus* on the native entomofauna, and in particular on the two native flies *A. aptera* and *C. moseleyi*, we used the abundance index recorded during the exhaustive geographical survey conducted from December 2004 to March 2006. We took into account the period of activity of the three species based on knowledge from the long-term biosurveillance programme for entomofauna running at Port-aux-Français; this consists of (a) three pitfall traps (Ø 9 cm, h 4 cm) opened for 5 d every 2–3 weeks, with the aim of collecting individuals of *M. soledadinus*, (b) one baited trap continuously operated targeting *A. aptera* (insects collected every 5–10 days), and (c) one yellow trap operated for 5 days every 2–3 weeks targeting *C. moseleyi*. Taking into account the seasonal patterns of activity of these species, we focused on the following pairwise comparisons: (1) *M. soledadinus* versus *A. aptera* (at least one of the two species present in the 338 records from coastal habitats, including seashore, under seaweeds, stones), (2) *M. soledadinus* versus A. *aptera* (at least one of the two species present in the 379 records from inland habitats, i.e. more than 50 m from the seashore, under stones and carrion), (3) *M. soledadinus* versus *A. aptera* (at least one of the two species present in the 155 records from carrion, along the seashore and inland), (iv) *M. soledadinus* versus *C. moseleyi* (at least one of the two species present in the 177 records from coastal habitats, including seashore, seaweeds and under stones).

Additional information on the impact of *M. soledadinus* on *A. aptera* and *C. moseleyi* was obtained from trapping results from a coastal site on Île Guillou in Golfe du Morbihan. On this island, three pitfall traps (Ø 9 cm, h 4 cm) were opened monthly for 5 days between January 1994 and July 2003, and then from March 2006 to November 2007. Collected insects were stored in 70% ethanol, identified to species level, and counted in the laboratory.

### Statistical analyses

GIS tools (ArcGIS 10.4, Esri) were used to map changes in the geographical distribution of *M. soledadinus* over time. The frequency distributions of the abundances of *M. soledadinus*, *A. aptera,* and *C. moseleyi* were represented in contingency tables, giving marginal (sum of each column, sum of each line) and grand (total number of individuals) totals. Expected frequencies were first computed from the totals assuming that there were no relationships between cells which would result in similar values between expected and observed frequencies. To assess differences among proportions, Chi-square tests were conducted (whenever classes with low frequencies occurred, frequencies from adjacent classes were pooled) as well as Fisher’s exact test (when pooling classes resulted in a 2 × 2 table). The analyses were conducted using Minitab 13 (Minitab Inc., State College, PA.).

## Data Availability

The datasets generated during and/or analysed during the current study are available from the corresponding author on reasonable request.
